# Editorial: Cognitive Impairment and Peripheral Neuropathy From Chemotherapy: Molecular Mechanisms and Therapeutic Approaches

**DOI:** 10.3389/fmolb.2022.962889

**Published:** 2022-07-15

**Authors:** Bojana Milutinovic, Anand Kumar Singh

**Affiliations:** ^1^ Department of Neurosurgery, MD Anderson Cancer Center, University of Texas, Houston, TX, United States; ^2^ Laboratory for Neuroimmunology, Symptom Research Department, MD Anderson Cancer Center, University of Texas, Houston, TX, United States

**Keywords:** chemotherapy, chemobrain, peripheral neuropathy, pain, cognition

## Introduction

Chemotherapy-induced neurotoxicity is a global health concern in the 21st century. According to the National Cancer Institute, by 2026, the US alone will have close to 20 million cancer survivors, with the majority of them having undergone chemotherapy, alone or in combination with surgery/radiotherapy (Miller et al., 2016). Neurotoxic side-effects of chemotherapy are common and may sometimes be severe. These side-effects include chemotherapy-induced peripheral neuropathy (CIPN) and chemotherapy-induced cognitive impairment (CICI). Acute CIPN and CICI may occur during treatment, but in many patients the symptoms of CICI and CIPN persist even after treatment is complete. Studies show that up to 75% of patients who are treated for cancer experience some form of CIPN or CICI (Taillibert et al., 2016). The symptoms of CIPN comprise numbness, tingling, burning pain, and hypersensitivity to temperature in the hands and feet. Mechanistically, CIPN is related to loss of intra-epidermal nerve (IENF) fibers, morphological and functional changes in mitochondria in dorsal root ganglia (DRG) and peripheral nerves (Maj et al., 2017; Ma et al., 2019). CICI is the result of long-lasting changes in the brain, where chemotherapeutics decrease brain connectivity, processing speed, impair attention, learning and memory, visual-spatial skills, multitasking, goal-directed behavior and disrupt fine motor functions (Tchen et al., 2003; Jim et al., 2012; Singh et al., 2022).

There is an unmet need to identify actionable targets to combat these severe neurotoxicities. Thus, the goal of this Research Topic is to collect cutting-edge studies exploring the molecular mechanisms of chemotherapy-induced neurotoxicities. This Research Topic of Frontiers in Molecular. Biosciences covers this important subject, with insightful reviews and original research articles.

## A bibliometric Approach to Iiterature Analysis Reveals Both Focused and Previously Unexplored Areas of Scientific Interest

In this systematic review, Corley and Allen used a bibliometric approach to mine and depict relevant information regarding cyclophosphamide, methotrexate, and fluorouracil (CMF)-induced cognitive dysfunction in breast cancer patients. The authors created term maps that demonstrate that the accumulation of knowledge in the initial decade (1990–1999) is characterized by poor differentiation of main terms and a high overlap of clusters. In contrast, in the following 2 decades, distinct term clusters formed and terms such as “inflammation” emerged. This is accompanied by periodic clustering of terms such as “apoptosis”, “oxidative stress” and “NF-kappa-b”. This type of research methodology can be applied to analyze continuous developments of a particular research area by gauging the terminology used in the publications based on journal output.

### The Brain Plays a Central Role in Chemotherapy-Induced Cognitive Impairments and in Peripheral Neuropathy

In this review, Orszaghova et al. have summarized the current literature on CICI, focusing on neurotoxic chemotherapy side effects in non-CNS cancer, including possible mechanisms and biomarkers. Factors contributing to the development of CICI include age, genetic predisposition, and psychological and sociodemographic background (Ahles and Root 2018). Animal model data suggest that the mechanisms of CICI include inhibition of hippocampal neurogenesis, myelin damage, excessive free radical production, inflammation and decreased vascularization and blood flow (Seigers et al., 2013). Additionally, in cancer patients who report cognitive dysfunction, inflammatory pathways (e.g., cytokine-cytokine receptor interaction, tumor necrosis factor signaling) are perturbed compared to patients that show no cognitive-related symptoms (Oppegaard et al., 2021). Dysregulation of the gut-microbiome by chemotherapy treatment may also contribute to CICI (Ciernikova et al., 2021).

The review by Omran et al. goes beyond peripheral nerves to understand the role of the brain in the pathophysiology of CIPN. This interesting review summarizes several studies that examined the interventions to the brain and spinal cord that caused or reduced CIPN. Some of the key features revealed by these studies are the hyperexcitability of the neurons in periaqueductal gray, thalamus, anterior cingulate cortex (ACC) and insular cortex during CIPN (Kleckner et al., 2017). The hyperexcitability in the brain is related to reduced GABAergic inhibition and alteration in the excitatory/inhibitory balance. Paclitaxel-induced CIPN is characterized by activation of astrocytes in the ACC following inflammation and neuronal 66 excitability (Leung and Cahill 2010; Masocha 2015). When key inflammatory pathways are blocked, GABA levels are restored, neuronal excitability is reduced, and consequently CIPN isresolved. Additionally, manipulating GPCR pathways to reduce PKC or MAPK phosphorylation in the brain reduces CIPN symptoms. Overall, these studies collectively show that CIPN is not exclusively a peripheral phenomenon and suggests that research should focus on the brain as the origin of CIPN.

## Increased Understanding of how Chemotherapy Affects Cerebral Blood Flow and Blood Flow Connectivity may Shed new Light on the Mechanisms of CICI

As previously mentioned, CICI is often observed as a consequence of treatment of non-CNS tumors. The two original research papers published in this issue focus on the effect chemotherapy has on blood vessels and blood flow and how it may affect cognitive function in both experimental models and cancer patients.

In the article by Groves et al., the authors used mouse models to study the effect of the common colon cancer therapeutics, 5-Fluorouracil (5-Fu)/leucovorin (LV), on cognitive function. Using a series of behavioral tests, the authors demonstrated that 5-FU/LV increases anxiety and reduces spatial memory retention in mice. While no microglial activation was observed, 5-Fu/LV-treated mice had a significant decrease in blood vessel length in the dorsal DG when compared with healthy controls. Blood vessel changes and disrupted blood flow are related to cognitive impairment (Zlokovic, 2008; Zlokovic, 2010).

In the second article, Zhang et al. used a novel approach in a first-of-its-kind study—arterial spin labeling perfusion magnetic resonance imaging to study cerebral blood flow changes in patients with non-small cell lung carcinoma. This is an especially important topic, as lung cancer accounts for approximately 11% of all cancer cases worldwide. Additionally, platinum compounds, which are highly correlated with CICI, are the first line of therapy in lung cancer (Siegers et al., 2013, Singh et al., 2020). Their results show that cerebral blood flow (CBF) and CBF connectivity are especially affected in the areas related to attention, including the superior temporal gyrus (STG), right middle frontal gyrus (MFG) and insula. Previously published data show a strong correlation between CBF changes in corresponding areas of the brain with reduced performance in the alerting and executive control attention networks in breast cancer patients (Tchen et al., 2003).
